# Modeling and measuring graduate students’ generative AI literacy: a study based on Marzano’s taxonomy

**DOI:** 10.3389/fpsyg.2026.1883978

**Published:** 2026-09-17

**Authors:** Yaozheng Zhi, Wei Yang, Kaili Huang

**Affiliations:** 1 School of Education, Shanxi Normal University, Taiyuan, Shanxi, China; 2 School of Chemical Engineering and Technology, Taiyuan University of Science and Technology, Taiyuan, Shanxi, China; 3 College of Public Administration, Huazhong University of Science and Technology, Wuhan, Hubei, China

**Keywords:** generative AI literacy, graduate students, measurement, model construction, scale development

## Abstract

**Background:**

Generative AI is rapidly reshaping graduate students’ academic research practices. Existing research lacks both literacy models specific to graduate students and a unifying theoretical framework to explain each dimension’s functional role and interrelationships, constraining its cultivation and evaluation.

**Methods:**

Semi-structured interviews with 14 professors were analyzed using grounded theory, with Marzano’s taxonomy of educational objectives serving as the theoretical framework for selective coding. A corresponding scale was developed and empirically validated with 308 graduate students through exploratory factor analysis, confirmatory factor analysis, and reliability and validity tests.

**Results:**

Five first-order dimensions and 15 s-order indicators were identified and validated through grounded theory analysis and questionnaire testing, with the scale demonstrating acceptable reliability and validity. Grounded theory analysis revealed that the five dimensions functioned as an interconnected whole. Cognitive foundation provided the underlying knowledge base for AI tool use; higher-order thinking critically evaluate and deeply processed AI-generated content; operational skills served as the practical tool that translated cognitive and critical thinking into academic outputs; metacognitive reflection monitored and evaluated the AI usage process; and ethical responsibility offered value guidance and normative boundaries for the functioning of all dimensions.

**Conclusion:**

This study developed and initially validated a scale for measuring graduate students perceived generative AI literacy. These findings offer actionable insights for designing AI-integrated graduate education and literacy cultivation strategies.

## 1 Introduction

The rapid advancement of generative AI is reshaping the ways in which academic knowledge is produced and disseminated. Large language models such as ChatGPT and DeepSeek have become widely integrated into graduate students’ academic workflows, including literature retrieval, code generation, paper revision, and research design (Liang et al., 2025; Partha, 2023). However, alongside these opportunities, notable challenges have emerged. Many graduate students use these tools at a superficial level, lacking a systematic understanding of underlying technical principles, the capacity for critical evaluation of AI-generated content, or a clear awareness of academic ethical boundaries (Henrik, 2023; Dai and Chan, 2026). More fundamentally, the field has yet to develop a systematic theoretical framework or a validated measurement instrument to address what constitutes generative AI literacy for graduate students (Chung, 2023). This may lead to risks such as technological misuse, academic misconduct, and a decline in higher-order cognitive capacities.

Existing research has explored generative AI literacy from three perspectives. In conceptual evolution, the field has moved from reactive AI literacy to discriminative AI literacy and then to generative AI literacy (Konishi, 2015; Andrew, 2024). In framework development, researchers have proposed multidimensional structures centered on cognition, practice, and ethics (Long and Magerko, 2020; Ng et al., 2021). In measurement, several assessment tools have been developed for college students (Liu et al., 2025; Zhang et al., 2025; Lee et al., 2026). However, two critical gaps remain. First, most existing frameworks adopt a generic “college student” label, overlooking the fact that graduate students differ substantially from undergraduates in terms of academic task complexity, research autonomy, and ethical responsibility. Few models have been tailored specifically to the graduate academic context. Second, existing literacy models tend to be enumerative. While frameworks cover multiple dimensions such as knowledge, skills, and attitudes, they lack a unifying theoretical principle to explain the distinct functional roles of each dimension and how they relate to one another. Therefore, this study adopts Marzano’s taxonomy of educational objectives as a theoretical organizing framework. It integrates interviews with grounded theory to construct a model of graduate students’ generative AI literacy and delineate its dimensions and interrelations. On this basis, a measurement scale was developed and empirically tested using exploratory and confirmatory factor analyses.

This study makes two theoretical contributions. On the one hand, it constructs a framework for graduate students, situating the dimensions with their actual academic practices and providing a theoretical basis for literacy cultivation. On the other hand, it establishes the applicability of Marzano’s taxonomy in this context, offering clear theoretical positioning for each dimension through the three-tier structure of the cognitive, metacognitive, and self-systems.

## 2 Literature review

### 2.1 Concept of generative AI literacy

Generative AI literacy has evolved from earlier notions of reactive and discriminative AI literacy. Following the formalization of the term “AI” at the Dartmouth Conference in 1956, early research centered on logical reasoning and natural language processing (Ankita et al., 2020). During this stage, AI largely operated within a reactive paradigm, relying on predefined rules without memory or learning capabilities (Skinner, 1958). Agre (1972) introduced the concept of AI literacy, emphasizing that students should understand the fundamental principles of intelligent technologies to support their learning. At this stage, literacy primarily referred to knowledge of algorithmic rules and data feedback mechanisms. However, due to limited computational power and technological constraints, reactive AI had minimal application in educational contexts, and the notion of AI literacy remained underdeveloped.

In the 1990s, advances in machine learning and neural networks facilitated a transition toward a discriminative paradigm. Discriminative AI enabled the analysis of learning behavior data and the generation of personalized recommendations (Pattyam, 2020). Building on this shift, Konishi (2015) redefined AI literacy as the capacity to critically reflect on and respond to the development of an intelligent society. Accordingly, discriminative AI literacy required not only operational proficiency but also an understanding of classification and decision-making mechanisms, as well as the ability to critically evaluate system-generated information.

The emergence of ChatGPT in 2022 marked a further transition toward the generative paradigm, in which AI systems function as creative agents rather than merely supportive tools. Generative AI demonstrated capabilities in content creation, natural language interaction, and complex reasoning, extending from information processing to higher-order cognitive support (Chiu et al., 2024). In this context, generative AI literacy was defined as a multidimensional psychological structure that enables individuals to effectively understand, apply, and evaluate generative technologies, while engaging in reflective consideration of their ethical implications (He et al., 2026). Such literacy ultimately supports a collaborative and synergistic relationship between humans and AI (Andrew, 2024; Aras, 2023; Dong et al., 2026).

AI literacy has evolved alongside technological advancements. The scope of AI literacy has expanded from algorithmic rules and classification mechanisms to content generation, human-AI collaboration, and ethical considerations. This trajectory suggests that literacy frameworks must be multidimensional, incorporating cognitive, operational, and ethical components rather than remaining confined to technical proficiency alone.

### 2.2 Framework construction of generative AI literacy

Currently, most theoretical constructions of generative AI literacy frameworks in academia are grounded in the concept of “competence,” typically structured around the dimensions of knowledge, skills, attitudes, and values. Some studies have emphasized top-level, generalizable frameworks. Long and Magerko (2020) proposed that generative AI literacy should encompass foundational knowledge, practical application abilities, and ethical awareness, organized across three core dimensions: cognition, practice, and ethics. Simms (2025) further argued that generative AI literacy extends beyond the critical evaluation of generated content to include a deeper understanding of underlying technical principles, as well as the responsible application of such knowledge and skills.

Other studies have developed more operationalized and hierarchical integrated frameworks. Ng et al. (2021) conducted a systematic review of 30 studies and identified four core dimensions: knowing and understanding, using and applying, evaluating and creating, and ethical issues. This work established a taxonomic foundation for subsequent research. Drawing on Marzano’s new taxonomy of educational objectives, Sun and Zhou (2025) constructed a two-dimensional integrated framework comprising three hierarchical systems and four progressive levels, highlighting literacy development as a gradual progression from foundational cognition to higher-order reflection. The three systems included the cognitive, practical, and perspective systems, while the four levels consisted of knowledge and comprehension, application and implementation, evaluation and creation, and ethics and safety. Wang et al. (2026a, 2026b) draw on the DIKW model and conceptualized literacy capabilities across four progressive levels, namely data operation, information processing, knowledge transformation, and wisdom decision-making, emphasizing a spiraling developmental logic from technical operation to value creation.

In summary, existing frameworks differ in their dimensional categorizations and hierarchical structures, a general consensus has emerged that generative AI literacy constitutes a multidimensional psychological structure, encompassing cognitive understanding, procedural skills, higher-order thinking, and ethical reflection. However, most frameworks have been developed for “college students” or “general learners” (Lin et al., 2025; Dogaru et al., 2025; Hou et al., 2025), and there remains a lack of literacy models specifically developed for the graduate students.

### 2.3 Indicator system construction of generative AI literacy

At the level of dimensional classification, although no unified standard has yet been established, knowledge cognition, operational skills, and ethical awareness are widely recognized as core components. Zhang et al. (2026) proposed that generative AI literacy among college students consisted of four first-level indicators, including theoretical knowledge, interactivity, application ability, and ethical awareness, together with 14 s-level indicators. Liu et al. (2025) focused on undergraduate student and developed a literacy assessment scale comprising five first-level dimensions, namely awareness awakening, knowledge construction, skill development, thinking cultivation, and ethical cognition, as well as 38 s-level indicators. Annapureddy et al. (2025) approached the issue from a dynamic perspective of human-AI interaction. They identified 12 core literacies, including technical understanding, prompt engineering, content evaluation, and ethical judgment.

In terms of assessment approaches, a comprehensive evaluation strategy that integrates quantitative and qualitative methods has gradually gained consensus. Lee et al. (2026) employed expert interviews, pilot testing, and large-scale empirical surveys to construct an AI literacy framework for college students, which included five first-level indicators, namely information and data literacy, communication and collaboration, digital content creation, safety and ethics, and problem solving. Zhang et al. (2025) developed the Generative AI Engagement Scale, which consisted of two subscales, frequency of use and interaction style, and applied multiple psychometric techniques, including classical test theory, exploratory factor analysis, confirmatory factor analysis, and item response theory. Yan et al. (2025) identified four first-level indicators of AI literacy, including awareness, use, evaluation, and ethics, through a combination of qualitative interviews and questionnaire surveys. This scale further incorporated characteristics specific to generative AI, particularly the ability to address risks such as misinformation, legal challenges, and systematic bias. Details are presented in Table 1.

**Table 1:** Comparison of existing generative AI literacy scales.

Researchers	Target population	First-level dimensions	Number of items	Theoretical basis
Zhang et al. (2026)	College students	Theoretical knowledge, interactivity, application ability, and ethical awareness	14	/
Liu et al. (2025)	College students	Awareness awakening, knowledge construction, skill development, thinking cultivation, and ethical cognition	38	/
Annapureddy et al. (2025)	General learners	Technical understanding, prompt engineering, content evaluation, and ethical judgment, among other 12 first-level dimensions	/	/
Lee et al. (2026)	College students	Information and data, communication and collaboration, digital content creation, safety and ethics, and problem solving	22	Digital competence framework
Zhang et al. (2025)	General learners	Self-interest-oriented use, and task-oriented use (use frequency subscale); questioningness, expressiveness, and preciseness (interaction style subscale)	20	Classical test theory, item response theory
Yan et al. (2025)	General learners	Awareness, usage, evaluation, and ethics	15	/

While these scales have laid a foundation for assessing generative AI literacy, most were developed for college students and fall short of capturing graduate students’ higher-order literacies in academic research. They also lack a theoretical framework capable of integrating the structural dimensions of generative AI literacy for this group.

### 2.4 Research gaps

Three major gaps can be identified. First, a unified conceptual understanding of graduate students’ generative AI literacy has yet to be established. Existing studies often apply generic labels derived from college students or general learners (Pandey et al., 2026; Jose et al., 2024), failing to fully capture the cognitive and metacognitive uniqueness of graduate students. Graduate students’ generative AI literacy should not only encompass general technological competencies but also be embedded in higher-order academic tasks such as literature critique, experimental design, and theoretical construction (Kang and Lee, 2026; Wu et al., 2026).

Second, existing frameworks for graduate students’ generative AI literacy lack a unifying theoretical principle. Most adopt an element-enumeration logic and lack a clear theoretical foundation to articulate the structural interrelations among dimensions and their functional roles (Chiu et al., 2024). A more theoretical approach is needed to construct a framework that reflects the academic task demands of graduate students, alongside operable measurement standards.

Third, the indicator construction and validation of graduate students’ generative AI literacy call for more precise research. Current assessment studies have primarily focused on college students as a whole (Wang et al., 2026a, 2026b), and the scales developed are insufficient to reflect the higher-order competency demands of graduate students in areas such as research innovation and academic writing. It is necessary to combine qualitative and quantitative methods to construct and validate core indicators of generative AI literacy specific to the graduate students.

## 3 Constructing a model of graduate students’ generative AI literacy

### 3.1 Research participants

This study selected university professors with experience in graduate education as interview participants in order to obtain expert insights for constructing a model of graduate students’ generative AI literacy. Participants were selected based on four criteria: they held the rank of professor or above, had at least 5 years of supervising master’s or doctoral students, possessed a working knowledge of generative AI, and came from diverse disciplines such as educational technology, computer science, and higher education.

The selection of professors as interview participants was based on the following considerations. First, professors were deeply involved in the full cycle of graduate academic training. They possessed systematic observation and judgment regarding students’ literacy needs, research design, and academic writing, and were able to offer comprehensive and structural insights that extended beyond individual student experiences. Second, the model-constructing phase addressed a normative theoretical question, namely what literacy graduate students ought to possess, rather than measuring their actual literacy levels. Addressing this question required professional judgment regarding academic training objectives and research competency expectations, which was precisely the strength of the professors. Third, the professors exhibited considerable heterogeneity in disciplinary coverage, years of supervisory experience, and academic perspectives. The diverse viewpoints from different disciplines could contribute to the theoretical saturation of the model.

From November to December 2025, the research team conducted consultative interviews with 14 professors from Double First-Class universities located in Shandong Province and Wuhan. The demographic characteristics of the participants are reported in Table 2.

**Table 2:** Basic information of the interviewees.

ID	Gender	Degree	Title	Discipline	Research field
S1	Male	Ph. D.	Professor	Science and engineering	Computer science
S2	Female	Ph. D.	Professor	Science and engineering	Computer science
S3	Male	Ph. D.	Professor	Humanities and social sciences	Educational technology
S4	Female	Ph. D.	Professor	Humanities and social sciences	Educational technology
S5	Male	Ph. D.	Professor	Science and engineering	Information science
S6	Female	Ph. D.	Professor	Humanities and social sciences	Higher education
S7	Male	Ph. D.	Professor	Science and engineering	Information science
S8	Male	Ph. D.	Professor	Science and engineering	Computer science
S9	Male	Ph. D.	Professor	Humanities and social sciences	Educational psychology
S10	Male	Ph. D.	Professor	Humanities and social sciences	Educational psychology
S11	Male	Ph. D.	Professor	Science and engineering	Information science
S12	Male	Ph. D.	Professor	Humanities and social sciences	Educational technology
S13	Male	Ph. D.	Professor	Science and engineering	Computer science
S14	Male	Ph. D.	Professor	Humanities and social sciences	Educational technology

### 3.2 Theoretical framework: Marzano’s taxonomy of educational objectives

In recent years, several frameworks comprehensively covered dimensions including knowledge, skills, and attitudes, they failed to clearly reveal the hierarchical relationships and functional mechanisms among the various constituent elements. Marzano’s taxonomy addressed this limitation through its three-layer structure–the cognitive, metacognitive, and self-system–which provided clear theoretical attribution and hierarchical positioning for literacy components. This study adopted Marzano’s taxonomy of educational objectives as a theoretical reference framework for the selective coding stage. Integrating the latest research findings in cognitive psychology, this taxonomy established an educational objective classification system oriented toward the cultivation of thinking skills (Marzano and Kendall, 2007). Its core contribution lay in breaking through the linear thinking limitations of Bloom’s taxonomy and systematically explaining the mechanism of learning behavior from the dual dimensions of knowledge domain and thinking processing level (Johnson et al., 2022).

In the knowledge dimension, Marzano categorized knowledge into three types: information, mental procedures, and psychomotor procedures. Information referred to declarative knowledge that answered the question of “what it is.” Mental procedures represented procedural knowledge addressing “how to do it.” Psychomotor procedures pertained to physical motor skills, ranging from basic movements to complex combined behaviors.

In the thinking processing, Marzano established a hierarchical framework comprising the self-system, the metacognitive system, and the cognitive system, which worked interactively to accomplish learning tasks. The self-system resided at the highest level, determining learners’ motivation and engagement by addressing the question of “whether one is willing to do it.” The metacognitive system occupied the middle level, undertaking the monitoring and regulation of cognitive processes to answer “how well it is done”. The cognitive system served as the foundational layer, encompassing four hierarchical cognitive operations from low to high: information retrieval, comprehension, analysis, and knowledge application, which addressed the question of “how to implement it specifically”.

It was noteworthy that two dominant theoretical paradigms exist in the field of educational objective taxonomy. One was the linear hierarchical model represented by Bloom, which divides cognitive objectives into six progressive levels from memory to evaluation. Another was the two-dimensional interactive model represented by Marzano, which emphasizes the non-linear combination of knowledge domains and processing levels. This study adopted Marzano’s framework over Bloom’s taxonomy for the following reasons. First, graduate students’ generative AI literacy involved three heterogeneous knowledge categories: information, mental procedure, and ethical attitude. Bloom’s unidimensional framework could not accommodate these three types of knowledge simultaneously, whereas Marzano’s knowledge domain dimension provided clear theoretical classification criteria. Second, although the revised edition of Bloom’s taxonomy had expanded the cognitive and knowledge dimensions to encompass information and mental procedures, its “cognitive–knowledge” structure still fell short of providing systematic theoretical grounding for metacognitive monitoring and ethical responsibility. In contrast, Marzano’s framework situated metacognitive reflection and ethics within its metacognitive system and self-system, respectively, offering clear theoretical positioning.

### 3.3 Qualitative data collection and analysis strategy

#### 3.3.1 Methodology

The qualitative data came from interviews with 14 university professors. Data analysis employed grounded theory coding procedures as an analytical tool to systematically code and categorize the interview materials. In the open and axial coding stages, the analysis followed the inductive logic of grounded theory, allowing all initial concepts and main categories to emerge progressively from the original narratives. In the selective coding stage, Marzano’s taxonomy of educational objectives was introduced as a theoretical reference framework to organize and interpret the structural relationships among the emerged categories. In other words, the Marzano framework was not introduced at the initial coding stage to constrain the analytical direction. Instead, it served as a structural reference to examine and integrate the emerged findings after the categories had largely taken shape. This strategy preserved the core spirit of grounded theory, which is generating from data, while enhancing the theoretical explanatory power of the model through late-stage theoretical referencing. The coding process strictly followed the systematic procedures of grounded theory. All categories emerged progressively from the original narratives rather than being directly classified according to a preset framework.

It should be noted that the selection of participants followed purposive sampling rather than the theoretical sampling required by grounded theory. Theoretical sampling emphasizes the iterative alternation between data collection and analysis until theoretical saturation is reached. In contrast, this study determined 14 participants at the sample selection stage based on explicit criteria. It employed grounded theory coding procedures as an analytical tool, without seeking to fully replicate all operational procedures of the methodology.

#### 3.3.2 Interview implementation and data collection

Semi-structured interviews were conducted from November to December 2025. The interviewees were recruited from Double First-Class universities in Shandong and Hubei Province, covering disciplines such as educational technology, computer science, and higher education. Each participant underwent a face-to-face interview lasting between 30 and 60 min, with an average duration of approximately 45 min. The interviews were conducted by two members of the research team. All interviews were audio-recorded and subsequently transcribed verbatim. Two members independently cross-checked the transcripts against the original recordings to ensure accuracy and completeness. Both the interviews and the subsequent analysis were conducted in Chinese; no translation procedures were involved.

The interview questions focused on three areas: the core literacies that distinguished graduate students who effectively used generative AI for high-quality research from general users; the relationships among these literacies and how they collectively supported academic innovation; Third, the most critical areas for strengthening graduate education to enhance generative AI literacy.

The questions served as the initial framework for the interviews. They were deliberately designed to be broad, allowing participant’s sufficient space to express their views without prematurely constraining the direction of their responses. Throughout the interviews, the research team posed targeted follow-up questions based on the participants’ answers to probe deeper into specific points. It ensured that concrete and analyzable qualitative materials were obtained while maintaining overall openness.

#### 3.3.3 Probing strategies and bias control

To ensure the collection of specific and analyzable qualitative data, the research team designed systematic probing strategies during the interviews. When the interviewees’ responses were relatively abstract, typical probes included: How exactly did this literacy manifest itself in graduate students’ daily academic work? Could you provide a specific example of a graduate student you had supervised who performed particularly well or encountered problems when using AI? How did these literacies you mentioned interact with each other in actual research practice? These probes were intended to elicit concrete behavioral events and observational cases, avoiding abstract judgments and vague generalities. All questions remained open-ended to prevent steering participants toward predetermined answers. All probes were naturally triggered by the interviewees’ responses, rather than being based on a preset theoretical framework or analytical dimensions.

#### 3.3.4 Coding quality

The coding work was led by the first author, with two doctoral students familiar with grounded theory forming the coding team. Prior to formal coding, the research team underwent a two-week training session covering the methodological principles of grounded theory, the operational procedures of the three-level coding, hands-on training with NVivo, and pilot coding exercises based on two non-interview transcripts. This training continued until an acceptable level of consistency was achieved among the three coders. Formal coding was conducted using NVivo software.

Disagreements during the coding process were resolved through collective discussion within the research team. For example, when a raw statement involved “ethics,” the coding team determined whether it referred to individual academic norms or social responsibility. Statements focusing on the specification of AI usage scope and adherence to academic integrity were categorized under “awareness of academic integrity”. Statements emphasizing the impact of AI on the academic ecosystem and concerns about educational equity were categorized under “awareness of academic social responsibility”. For statements that could not be clearly classified, the coding team marked them as “pending,” returned to the original text to re-examine the contextual meaning, and compared them with similar categories until consensus was reached. All coding decisions were documented to maintain a trail.

In addition, the research team held regular reflexive meetings to critically examine potential preconceptions that may have emerged during the coding process, with particular attention to whether the presence of the Marzano framework had unintentionally influenced coding judgments regarding the raw materials.

#### 3.3.5 Ethical approval and informed consent

All participants signed informed consent forms prior to the interviews, in which they were informed of the research purpose, data usage procedures, privacy protection measures, and their right to withdraw voluntarily. All interview recordings and transcripts were stored in de-identified form and were accessible only to core members of the research team.

### 3.4 Indicator construction: three-level coding in grounded theory

#### 3.4.1 Open coding

This study followed established grounded theory procedures, using “graduate students’ generative AI literacy” as the central analytical node. Among the 14 interview transcripts, 12 were selected for detailed line-by-line analysis, comprising approximately 120,000 words in total. The coding process was conducted in two main steps:

Initial concepts were refined through repeated comparison, analysis, and synthesis of all labels. Labels with different expressions but similar connotations were merged into the same concept. The merging followed the principle of combining like items, using the identity of the psychological behaviors or ability requirements as the criterion. For example, labels such as working principles of large language models, understanding how AI learns, and inherent limitations of AI technology all pointed to systematic cognition of generative AI principles, and were therefore merged into the initial concept of “understanding of technical principles”. Through this step, the 329 raw labels were condensed into 96 initial concepts. Partial coding results are presented in Table 3.

**Table 3:** Exemplary results of open coding.

Category	Raw data
Working principles of large language models	Graduate students must first understand how large language models work; they cannot simply use them without knowing the principles. (S1)
Understanding how AI learns	Understanding how AI learns and is trained is very important for graduate students; otherwise, it is difficult to judge the logic behind AI-generated content. (S11)
Functional features of AI software	Graduate students need to understand the functional features of different AI software in order to make reasonable choices based on their research task requirements. (S2)
Comparison of tool strengths and weaknesses	I often ask my students to compare the strengths and weaknesses of different AI tools. For example, ChatGPT is stronger in text generation and logical reasoning, while DeepSeek has an advantage in processing Chinese literature and long texts. (S8)
Applicability to theoretical framework construction	Graduate students must know which tasks are suitable for AI and which are not. For instance, when constructing a theoretical framework, AI can be used to broaden thinking, but the final judgment must be made by the students themselves. (S6)
Role-playing prompts	When designing prompts, I ask my graduate students to try having AI play a specific role, such as you are a reviewer, so that the generated content better meets their needs. (S4)
Guiding AI to progress incrementally	I tell my graduate students not to expect AI to give a perfect answer in one go. They need to guide it step by step, such as asking for a general framework first, then inquiring about specific details. (S5)
Content organization	AI-generated content is often fragmented. Graduate students must first extract the useful information and then reorganize it logically; they cannot simply copy and paste. (S2)
Accuracy judgment	Graduate students must have the ability to judge whether AI-generated content is correct. They should not blindly trust it just because it sounds fluent. Key data and conclusions must be verified by themselves. (S3)
Avoiding plagiarism of AI content	AI-generated content cannot be directly inserted into a paper. It must be understood and internalized by the students, and they must indicate which parts of the paper were generated with the assistance of AI. (S8)
Human-AI task allocation	Graduate students need to know which tasks can be accomplished more efficiently with AI and which must be done by themselves. For example, data analysis can be assisted by AI, but research conclusions must be drawn by the students themselves. (S2)
Breaking through fixed thinking patterns	When graduate students encounter stagnation in their research, they can use AI to obtain heuristic analytical frameworks and entry points from multidisciplinary perspectives, thereby breaking through existing cognitive limitations. (S1)
Correcting AI misunderstanding	AI often misunderstands what users mean. When this happens, users need to rephrase the question and ask again until the AI understands correctly. (S5)
Awareness of consequences of improper use	Graduate students must be aware of the consequences of misusing AI, such as paper rejection, being identified as having committed academic misconduct, or even affecting their degree. (S14)

It should be noted that Table 2 presents only representative examples of each initial concept to illustrate the basic logic of the coding process. For a small number of quotations with ambiguous boundaries or multiple possible interpretations, the coding team marked them as “pending” and returned to the original text to re-examine their contextual meanings. Through repeated comparison with similar categories, the final classification was determined, ensuring that all statements were categorized with justifiable grounds. Because these ambiguous cases were handled following the same coding rules and were all clearly classified in the end, they are not separately presented.

#### 3.4.2 Axial coding

The research team repeatedly compared and reorganized the 96 initial concepts derived from the open coding stage. By analyzing the roles these concepts played in graduate students’ generative AI literacy and their interrelationships, they were integrated and summarized into main categories. Categorization followed the principle of functional grouping: initial concepts serving the same literacy function were grouped into the same category, with category names capturing the common attributes of subordinate concepts. For example, “understanding of technical principles, tool cognitive competence, and identification of application scenarios” all served the function of systematic cognitive reserve and were grouped into the “cognitive foundation” category.

For concepts that could belong to multiple categories, the coding team adopted “core functional orientation” as the boundary criterion, determining the primary function of the concept within the literacy structure. For example, although “critical thinking for verification” involved cognitive analysis activities, its core function lay in making value judgments and distinguishing truth from falsehood regarding AI outputs, rather than the regulation of one’s own cognitive processes. It was therefore classified under “higher-order thinking” rather than “metacognitive reflection.” Concepts that could not be clearly classified were marked as “boundary cases” and were determined by comparing their similarities and differences with typical concepts of adjacent categories, and the decisions were recorded.

Through the above strategies, the 96 initial concepts were integrated into 15 main categories (Table 4), achieving an analytical progression from concepts to categories.

**Table 4:** Axial coding results.

Core category	Corresponding initial concepts
Understanding of technical principles	Working principles of large language models, understanding AI can make errors, clarifying the timeliness of AI knowledge, understanding how AI learns, inherent limitations of AI technology
Tool cognitive competence	Functional features of AI software, comparison of tool strengths and weaknesses, practical boundaries of AI tools
Identification of application scenarios	Applicability to literature retrieval, applicability to data cleaning, applicability to paper polishing, applicability to topic ideation, applicability to research design, applicability to theoretical framework construction
Prompt design competence	Role-playing prompts, task description design, output format setting, chain-of-thought prompts, iterative optimization of prompts, step-by-step guidance, providing examples, setting response perspective, constraining response scope, reverse questioning
Multi-turn dialogue competence	Clarification through follow-up questions, adjusting questions based on responses, guiding AI to progress incrementally, correcting AI misunderstandings, summarizing key points of dialogue, switching discussion angles, saving effective dialogue chains, identifying dialogue dead ends and redirecting
Output processing competence	Content organization, language polishing, visual presentation, format standardization, information filtering and deduplication, logical restructuring and optimization, integration with one’s own ideas, labeling AI-generated content
Critical thinking for verification	Accuracy judgment, identification of content bias, not blindly trusting AI conclusions, identification of argumentative flaws, scrutiny of conceptual appropriateness, checking the integrity of reasoning chains, identifying underlying assumptions and questioning them, comparative analysis of different perspectives, detection of factual errors, identification of fabricated citations, detection of temporal errors, verification of literature sources, identification of data inconsistencies, judgment of commonsense plausibility, follow-up questioning, reverse verification
Research hypothesis generation thinking	Broadening thinking with AI, discovering interdisciplinary connections, generating new ideas, breaking through fixed thinking patterns, generating innovative hypotheses
Human-AI collaboration thinking	Human-AI task allocation, collaboration process design, balance between autonomy and AI, leveraging respective strengths, evaluation of collaboration effectiveness
Monitoring of use process	Awareness of proper operation, detection of biased thinking, checking for over-reliance, awareness of cognitive inertia
Academic outcome evaluation	Judgment of efficiency improvement, assessment of output quality, analysis of gains and losses with AI assistance, reflection on usage effectiveness
Strategic transfer and adaptation	Cross-task application of experience, adjustment of strategies based on tasks, flexible adaptation of methods, exchange of strategies across different tools, context identification and strategy matching
Awareness of academic integrity	Specification of AI usage scope, avoiding plagiarism of AI content, adherence to academic norms, clarifying what is acceptable and what is not, awareness of consequences of improper use, self-checking and quality control
Awareness of academic social responsibility	Impact of AI on the academic ecosystem, concern for educational equity, responsibility to the academic community, digital divide in AI usage, participation in the development of AI academic norms
Awareness of knowledge contribution attribution	Distinguishing human-AI contributions, attribution of AI-generated outcomes, proper authorship, respect for intellectual property, consideration of AI contribution in academic evaluation

#### 3.4.3 Selective coding

The research team repeatedly compared and logically analyzed the 15 main categories. By examining their hierarchical relationships, functional complementarities, and correspondences with graduate academic work stages, five higher-order aggregations were identified, corresponding to the five functional domains: cognitive foundation, operational skills, higher-order thinking, metacognitive reflection, and ethical responsibility. The formation of these five domains was entirely driven by the logical relationships among the categories, without any external theoretical framework.

The five functional domains were then mapped onto the three structures of Marzano’s taxonomy. Cognitive foundation, operational skills, and higher-order thinking fell within the cognitive system; metacognitive reflection corresponded to the metacognitive system; and ethical responsibility was situated in the self-system. The mapping followed these principles: categories primarily involving knowledge acquisition, skill execution, or thinking processes were assigned to the cognitive system; those concerning the monitoring and evaluation of one’s own cognitive activities were assigned to the metacognitive system; and those involving value beliefs, motivational judgments, or the internalization and enactment of social norms were assigned to the self-system. In other words, the five dimensions were identified from the categorical relationships within the data, while the Marzano framework served as a theoretical language to interpret the emergent structure, rather than predetermining the number or content of the dimensions. The structure and its interrelations are presented in Table 5.

**Table 5:** Selective coding results.

Selective category	Axial category	Mapping to Marzano’s framework	Dimensional interpretation
Cognitive foundation	Understanding of technical principles	Cognitive system: information retrieval–information	This dimension represented the cognitive reserve that underpinned graduate students’ ability to conduct generative AI-assisted academic work. It emphasized systematic understanding of AI technical principles, tool capabilities, and application scenarios, ensuring that graduate students could accurately judge when and to what extent to employ AI in their academic work.
	Tool cognitive competence	Cognitive system: comprehension–information	
	Identification of application scenarios	Cognitive system: comprehension–information	
Operational skills	Prompt design competence	Cognitive system: comprehension–mental procedures	This dimension represented the systematic action capability that transformed cognitive foundations into tangible academic productivity. It emphasized graduate students’ ability to design effective prompts, engage in multi-turn dialogues, and process AI-generated content.
	Multi-turn dialogue competence	Cognitive system: comprehension–mental procedures	
	Output processing competence	Cognitive system: comprehension–mental procedures	
Higher-order thinking	Critical thinking for verification	Cognitive system: analysis–mental procedures	This dimension represented the core mental model through which graduate students engaged in higher-order cognitive processing. It constituted a thinking cycle of “detecting factual errors–generating research hypotheses–collaborative execution”, collectively driving the deep integration of academic cognition and practical competencies.
	Research hypothesis generation thinking	Cognitive system: knowledge utilization–mental procedures	
	Human-AI collaboration thinking	Cognitive system: knowledge utilization–mental procedures	
Metacognitive reflection	Monitoring of use process	Metacognitive system: Metacognitive monitoring	This dimension represented graduate students’ self-regulation capability in using generative AI. It emphasized maintaining cognitive autonomy in academic work, including timely monitoring of the usage process, objective evaluation of usage outcomes, and adaptive transfer of effective strategies to new academic contexts.
	Academic outcome evaluation	Metacognitive system: Metacognitive monitoring	
	Strategic transfer and adaptation	Metacognitive system: Metacognitive monitoring	
Ethical responsibility	Awareness of academic integrity	Self-system: self-regulation	This dimension represented the spiritual pillar that enabled graduate students to uphold the bottom line of academic ethics when using generative AI. It emphasized that while enjoying the convenience of technology, graduate students must always clarify what could be done, what should be done, and what must be done by themselves.
	Awareness of academic social responsibility	Self-system: self-regulation	
	Awareness of knowledge contribution attribution	Self-system: self-regulation	

### 3.5 Theoretical saturation test

To ensure the rigor and reliability of the constructed indicators, a coder consistency test was first conducted. Two doctoral students familiar with grounded theory, who were not involved in the initial coding process, were invited to independently code approximately 20% of the interview transcripts (around 25,000 words) under the guidance of the research team. During coding, discrepancies arose due to variations in respondents’ disciplinary backgrounds and research experiences, which led to differences in the emphasis of their statements. All disagreements were listed and examined through collective discussion. These cases were found to reflect only surface-level differences in wording, with core meanings remaining classifiable under existing concepts or categories; no new codes were required. Consensus was reached on all items through discussion. Given the small sample size, Krippendorf’s α was used as the primary reliability statistic instead of confidence intervals. The α coefficients for initial concepts and main categories were 0.820 and 0.860, respectively, indicating satisfactory objectivity and reproducibility of the coding.

A theoretical saturation test was conducted. Two complete interview transcripts from distinct disciplinary backgrounds were reserved and excluded from the main coding process to avoid homogeneity bias. Theoretical saturation was defined by the criterion that two consecutive reserved transcripts would yield no new initial concepts or core categories, nor any additional properties within existing categories. The research team applied the same three-level coding procedure to the two reserved transcripts, documenting any potential new concepts, categories, or properties, and compared them against the established coding framework. No new significant concepts or categories emerged, no additional properties were identified, and the relational structure among categories remained stable. The two reserved transcripts provided only corroborative evidence without extending existing categories or revising dimensional logic. Further data collection would therefore only replicate existing information with diminishing marginal returns, and no additional qualitative sampling was necessary.

## 4 Model establishment of graduate students’ generative AI literacy

### 4.1 Graduate students’ generative AI literacy structure

#### 4.1.1 Cognitive foundation

Cognitive foundation referred to the knowledge base that graduate students established regarding generative AI. Within Marzano’s framework, the “information retrieval” and “comprehension” levels of the cognitive system involved the acquisition and processing of declarative knowledge (Marzano and Kendall, 2007). Understanding technical principles, tool cognitive competence, and identifying application scenarios aligned with the “information retrieval” and “comprehension” levels of the cognitive system, addressing the question of “what graduate students need to know”.

Understanding technical principles meant that graduate students could explain the basic working mechanisms of large language models, identify potential logical fallacies or factual inaccuracies in AI outputs. As graduate academic work heavily relied on information accuracy and reasoning rigor, establishing a systematic understanding of the underlying technical principles was crucial. “It enabled them to rationally judge the credibility and accuracy of AI outputs in core academic activities such as literature critique, theoretical construction, and model building” (S1).

Tool cognitive competence referred to graduate students’ ability to identify the main functions and suitable application scenarios of different AI tools, distinguish their relative strengths in tasks such as text generation and literature processing, and make targeted selections based on specific academic tasks (Razvan et al., 2023). “Given the high diversity and contextual dependence of academic tasks, graduate students needed to move beyond reliance on a single tool, establish precise knowledge of different tool capabilities, and make optimal selections among multiple AI tools based on specific academic tasks” (S8).

Identifying application scenarios referred to graduate students’ ability to distinguish which academic tasks could be accomplished more efficiently with AI assistance and which had to be completed independently by researchers, and to plan the extent of AI involvement in the research process accordingly. Graduate academic activities covered multiple stages including literature retrieval, data cleaning, paper polishing, topic ideation, and research design, with significant variations across different tasks in the degree and manner of AI reliance. “It was essential to establish a clear awareness of AI application scenarios” (S2).

#### 4.1.2 Operational skills

Operational skills referred to graduate students’ ability to translate cognitive foundations into academic productivity. Within Marzano’s framework, the “comprehension” level of the cognitive system involved the mastery and execution of procedural knowledge (Marzano and Kendall, 2007). Prompt design competence, multi-turn dialogue competence, and output processing competence, respectively, pointed to graduate students’ application and execution of mental procedure knowledge during AI interactions. These corresponded to the “comprehension” level of the cognitive system, addressing the question of “what graduate students need to do”.

Prompt design competence was defined as graduate students’ ability to formulate effective prompting strategies based on research tasks, so as to precisely guide AI in generating outputs that met academic requirements (Wang et al., 2025). “It included assigning appropriate academic roles to AI according to the nature of the research task, translating vague research intentions into clear and structured task instructions, and specifying the format, length, and level of detail of the output content” (S4).

Multi-turn dialogue competence required graduate students to possess the operational skills for sustained, iterative interaction with generative AI. The initial outputs of AI often fell short of the complex demands of academic research, requiring iterative refinement through follow-up questions, rephrased prompts, progressive deepening, and correction of misunderstandings. “Optimal output was rarely achieved in a single attempt; rather, it had to be gradually elicited through sustained multi-turn dialogue” (S5).

Output processing competence referred to graduate students’ ability to deeply process and integrate AI-generated content, including filtering, deduplication, logical reorganization, and structural optimization, and to synthesize it with their own academic perspectives. “AI-generated information was often fragmented. Only through deep processing and integration by researchers could it truly serve academic production” (S6).

#### 4.1.3 Higher-order thinking

Higher-order thinking meant that the core cognitive model through which graduate students achieved cognitive breakthroughs and knowledge innovation in human-AI collaboration. Within Marzano’s framework, the cognitive system comprised four hierarchical levels of cognitive operations: information retrieval, comprehension, analysis, and knowledge application (Marzano and Kendall, 2007). Critical thinking for verification, research hypothesis generation thinking, and human-AI collaboration thinking corresponded to the “analysis” and “knowledge application” levels of the cognitive system. Together, they addressed the question of “how graduate students achieve innovation with AI assistance”.

Critical thinking for verification required graduate students to comprehensively judge and verify content generated by AI (Athaluri et al., 2023). This involved assessing its accuracy and reliability through multi-source comparison, follow-up verification, and reverse examination, and then deciding whether to accept, revise, or reject the information provided by AI (Matthew, 2022; Costa et al., 2024). “Graduate students should cultivate this capacity in their academic research” (S14).

Research hypothesis generation thinking referred to the mode of thinking through which graduate students used generative AI to break through fixed thinking patterns. “This meant that when research reached an impasse, graduate students could draw on AI to obtain interdisciplinary analytical frameworks, integrate multiple sources of information into testable hypotheses, and form independent judgments based on AI-generated suggestions” (S1).

Human-AI collaboration thinking guided the approach through which graduate students determined the division of labor between human and AI based on the cognitive complexity and creative demands of tasks, and adjusted collaboration strategies accordingly. The degree of reliance on AI varied significantly across different academic tasks. “It was necessary to clarify which tasks are suitable for AI assistance and which had to be completed independently by graduate students” (S13).

#### 4.1.4 Metacognitive reflection

Metacognitive reflection referred to graduate students’ ongoing monitoring, evaluation, and regulation of their own cognitive processes and behavioral strategies when using generative AI. Within Marzano’s framework, the metacognitive system was defined as the psychological mechanism responsible for monitoring and regulating cognitive processes (Marzano and Kendall, 2007), and its core function was “metacognitive monitoring.” Monitoring of the use process, academic outcome evaluation, and strategic transfer and adaptation all pointed to graduate students’ continuous examination and optimization of their AI usage behaviors. These theoretically corresponded to the metacognitive monitoring function of the “metacognitive system,” addressing the question of “how well graduate students perform”.

Monitoring of the use process required graduate students to maintain cognitive vigilance while using generative AI, continuously monitoring their own operational strategies and thinking states. This included recognizing whether they were over-relying on AI outputs or being unconsciously steered by AI, and making timely adjustments to preserve cognitive autonomy. “Graduate students needed to develop this reflective literacy in order to maintain academic initiative while benefiting from technological convenience” (S6).

Academic outcome evaluation meant that graduate students could objectively assess the actual effectiveness of generative AI-assisted academic work. It involved evaluating the gains and losses of AI use across multiple dimensions, including time investment, output quality, and cognitive load, and determining the applicability of AI tools to specific academic tasks accordingly. “Such evaluation helped graduate students establish a reliable basis for subsequent strategy optimization” (S13).

Strategic transfer and adaptation meant that graduate students could identify structural similarities across different academic tasks, transfer effective strategies developed in one context to new task situations, and make flexible adjustments based on actual feedback. For example, “prompting methods developed in literature review might also be applicable to research design” (S5).

#### 4.1.5 Ethical responsibility

Ethical responsibility referred to the value judgments and behavioral norms that graduate students upheld regarding academic integrity, social impact, and knowledge contribution attribution when using generative AI. Within Marzano’s framework, the self-system was defined as the mechanism that determines whether an individual initiates, maintains, or terminates a particular task. Its core functions included “beliefs and value judgments” and “self-regulation” (Marzano and Kendall, 2007). The self-system fundamentally shaped learners’ dispositions toward specific tasks–determining whether a task was worth pursuing and under what value premises–and thereby governed their willingness to invest cognitive and behavioral resources. Ethical responsibility corresponded to the self-system. This was because awareness of academic integrity, social responsibility, and knowledge contribution attribution collectively embodied graduate students’ value judgments regarding appropriate AI use, while also reflecting their recognition of the broader consequences of their academic conduct. Such beliefs and value orientations were precisely the manifestations of the “beliefs and value judgments” and “self-regulation” functions.

Awareness of academic integrity referred to graduate students’ strict adherence to academic norms when using AI, and their clear understanding of the essential difference between AI-assisted work and AI-generated content (Liebrenz et al., 2023). “In other words, the scope and extent of AI usage needed to be clearly indicated. Graduate students needed to be aware of the consequences of improper use” (S7).

Awareness of academic social responsibility meant that graduate students could recognize the far-reaching impact of generative AI on the academic ecosystem and social development, including inequalities in academic resource distribution and the widening digital divide among different groups (Feng and Lyu, 2025). “As members of the academic community, graduate students needed to assume corresponding social responsibilities, actively participate in the development of AI academic norms, and promote the responsible application of technology” (S3).

Awareness of knowledge contribution attribution required graduate students to clarify the boundaries of responsibility between humans and AI in knowledge production. “Graduate students needed to clearly distinguish the respective contributions of humans and AI in the research process, and indicate in the paper which stages were assisted by AI” (S9). At the same time, they should reasonably consider the proportion of AI-assisted work in academic evaluation, ensuring the accuracy of knowledge contribution attribution.

### 4.2 Graduate students’ generative AI literacy model

The five core dimensions and their interrelationships could be elaborated as follows:

Cognitive foundation provided knowledge-based support for graduate students’ effective use of generative AI. The depth of graduate students’ understanding of AI technology directly affected the implementation of operational skills, the quality of higher-order thinking, and the effectiveness of metacognitive monitoring, while also informing ethical judgments. “Understanding technical foundation was essential for conducting high-quality research” (S6).

Operational skills served as the action role that translated cognitive foundations into academic productivity. They not only refined and expanded the cognitive foundation through practice, but also fed back into the development of higher-order thinking. “Without proficiency in prompt design, multi-turn interaction, and content integration, research ideas could hardly be executed effectively by AI” (S4).

Higher-order thinking guided graduate students beyond instrumental use of AI, enabling deeper knowledge integration and cognitive breakthroughs in human-AI collaboration. Its effective deployment enhanced the precision of strategy adjustments in practice, deepened metacognitive reflection, and informed ethical judgments. “Without it, graduate students’ academic creativity would be constrained” (S12).

Metacognitive reflection played a monitoring and regulatory role in the process of AI use. Monitoring of the use process, academic outcome evaluation, and strategic transfer and adaptation enabled graduate students to evaluate usage effectiveness, and transfer effective strategies to new academic contexts. “Without it, graduate students would struggle to balance technological convenience with cognitive autonomy” (S6).

Ethical responsibility provided value guidance and normative boundaries for the application of cognitive foundation, operational skills, higher-order thinking, and metacognitive reflection, cutting across all four dimensions. “The absence of ethical awareness would lead AI use to deviate from academic norms and undermine the credibility of research” (S7).

The above five dimensions functioned as an interconnected whole. Cognitive foundation provided the knowledge base for the entire system. Higher-order thinking was responsible for critically evaluating and deeply processing AI-generated content. Operational skills served as the practical tool that translated cognitive and critical thinking capacities into academic outputs. These three dimensions collectively corresponded to Marzano’s cognitive system, embodying a process from knowledge accumulation to action execution and then to cognitive breakthrough. Metacognitive reflection continuously performed monitoring, evaluation, and regulation functions, which precisely reflected the regulatory role of Marzano’s metacognitive system over the cognitive system. Ethical responsibility corresponded to Marzano’s self-system, providing value guidance and normative direction for the operation of all dimensions.

## 5 Measurement of graduate students’ generative AI literacy model

### 5.1 Scale development

The scale items were initially developed based on the 15 s-level indicators of graduate students’ core generative AI literacy derived from grounded theory analysis. It should be noted that the generative AI literacy measured by the scale referred to graduate students perceived and self-reported levels of the five dimensions, rather than their actual performance ability measured through objective tasks. To ensure content validity, one professor specializing in psychometrics was invited to evaluate the items for accuracy and clarity, and to revise any ambiguously worded items. Subsequently, two professors in higher education (who were not interview participants) were invited to review the revised items for comprehensibility and alignment with the core constructs of generative AI literacy. Further refinements were made based on their feedback, resulting in a preliminary scale comprising 15 items (Table 6). To quantify the correspondence between the items and their intended dimensions, the same three experts independently rated the relevance of each item to its assigned dimension on a 4-point scale (1 = not relevant, 4 = highly relevant). Both the item-level content validity index (I-CVI) and the scale-level content validity index (S-CVI) were calculated. The I-CVI values for the 15 items ranged from 0.870 to 1.000, with a universal agreement S-CVI of 0.873 and an average S-CVI of 0.941, indicating acceptable content validity.

**Table 6:** Items of the graduate students’ generative AI literacy scale.

Dimension	Secondary dimension	Item
Cognitive foundation	Understanding of technical principles	I understand the basic working principles of large language models, as well as their inherent limitations in factual generation, logical reasoning, and knowledge representation.
	Tool cognitive competence	I can compare the functional features, strengths, and weaknesses of different generative AI tools, and make reasonable choices based on my academic task requirements.
	Identification of application scenarios	I can accurately determine the scope of generative AI use in academic work, and recognize the differences in the degree of AI reliance across various tasks.
Operational skills	Prompt design competence	I can design effective prompts, such as setting AI’s academic role, describing research tasks, specifying output format, and guiding AI to reason step by step.
	Multi-turn dialogue competence	I can engage in in-depth interaction with generative AI through multi-turn questioning, rephrasing inquiries, and correcting AI’s misinterpretations of my intentions.
	Output processing competence	I can organize, restructure, and optimize AI-generated content, and integrate it organically with my own ideas.
Higher-order thinking	Critical thinking for verification	I can critically evaluate AI-generated content, including its logical soundness, argumentative rigor, potential biases, and factual reliability.
	Research hypothesis generation thinking	I can use generative AI to broaden my research perspectives, discover interdisciplinary connections, and generate innovative research hypotheses.
	Human-AI collaboration thinking	I can reasonably design the division of labor between human and AI, clarifying which tasks should be assisted by AI and which should be led by myself.
Metacognitive reflection	Monitoring of use process	When using generative AI, I can monitor whether my operations are appropriate, and guard against over-reliance or uncritical acceptance of AI output.
	Academic outcome evaluation	I can objectively evaluate the actual effectiveness of generative AI in supporting my academic work, in terms of time investment, output quality, and effort involved.
	Strategic transfer and adaptation	I can transfer AI use strategies developed in one academic task to other task contexts, and adjust them flexibly according to the actual situation.
Ethical responsibility	Awareness of academic integrity	When using generative AI, I strictly adhere to academic norms, clearly specify the specific stages and scope of AI assistance in my papers, and refrain from academic misconduct such as data fabrication and plagiarism.
	Awareness of academic social responsibility	I pay attention to the impact of generative AI on the academic ecosystem and social equity, and am willing to actively participate in discussions and initiatives regarding AI academic norms.
	Awareness of knowledge contribution attribution	I can clearly distinguish between human and AI contributions in knowledge production, clearly specify which parts were AI-assisted, and respect intellectual property rights.

A key design choice was to assign a single item to each secondary indicator. This decision was informed by two considerations: the grounded theory analysis had already provided substantial qualitative specification of the 15 indicators through three-level coding, with each carrying clear theoretical meaning and behavioral referents. The scale was developed to provide initial quantitative validation of the grounded-theory literacy structure, specifically to examine whether the five first-order dimensions could be empirically confirmed. In this regard, the 15 items serve as representative measures of their respective secondary indicators, providing preliminary evidence for the theoretical structure rather than serving as comprehensive psychometric assessments of each secondary construct.

The scale employed a 5-point Likert response format, ranging from 1 (strongly disagree) to 5 (strongly agree). All items were positively worded, with no reverse-scored items. The scale was administered in Chinese and no translation were involved.

### 5.2 Scale validation

#### 5.2.1 Questionnaire distribution and collection

In January 2026, the research team distributed electronic questionnaires via the Wenjuanxing platform to full-time graduate students from nine universities in Shanxi and Hubei provinces, including five “Double First-Class” universities and four regular universities. The questionnaires were disseminated through graduate class groups by collaborating faculty members from each institution. The survey was conducted anonymously, and all data were used solely for academic research. Eligibility criteria for participants included: current full-time graduate student status, at least 3 months of experience using generative AI tools, and informed consent to voluntary participation. A total of 317 questionnaires were collected, of which 9 were excluded: 5 were invalid due to excessively short completion time (under 120 s) and patterned response patterns, and 4 were invalid because over 80% of items received the same response. A total of 308 valid questionnaires were obtained, accounting for 97.16% of the collected responses. Due to the low proportion of missing data (below 1%), no imputation was performed. The demographic characteristics of the participants are presented in Table 7.

**Table 7:** The characteristics for graduate students.

Category	Subcategory	Frequency	Percentage (%)
Gender	Male	246	79.87
	Female	62	20.13
Academic discipline	Science and engineering	68	22.08
	Humanities and social sciences	132	42.86
	Medicine	56	18.18
	Agriculture	22	7.14
	Interdisciplinary	30	9.74
Degree level	Master’s degree students	265	86.04
	Doctoral degree students	43	13.96
University type	“Double First-Class” universities	168	54.54
	Other universities	140	45.45

To facilitate both exploratory and confirmatory factor analyses, the 308 valid questionnaires were randomly split into two subsamples of 154 cases each. Tests of demographic differences between the two subsamples revealed no significant differences in gender (*χ*^2^ = 0.12, *p* = 0.732), disciplinary background (*χ*^2^ = 1.89, *p* = 0.596), degree level (*χ*^2^ = 0.43, *p* = 0.512), or institution type (*χ*^2^ = 0.56, *p* = 0.454), indicating good comparability between the two subsamples and satisfying the data requirements for subsequent factor analyses.

#### 5.2.2 Exploratory factor analysis

To examine the scale’s structure, 154 valid questionnaires were randomly selected from the total sample and designated as Dataset A for exploratory factor analysis. The suitability of the data for factor analysis was first assessed using the Kaiser-Meyer-Olkin (KMO) measure and Bartlett’s test of sphericity. The KMO value was 0.874, indicating sufficient common variance among items. Bartlett’s test of sphericity was significant ( $x^{2}$ = 1892.567, *p* < 0.001), supporting the appropriateness of factor analysis.

Principal component analysis was conducted with varimax rotation to extract factors with eigenvalues greater than 1. This method, as a commonly used extraction approach in exploratory factor analysis, effectively identifies the underlying structural patterns among observed variables and demonstrates good stability with relatively limited sample sizes (Fabrigar et al., 1999). Items were retained based on two criteria: factor loadings above 0.600 on their respective factors, and theoretical consistency within each factor. All 15 initial items met these criteria and were retained. A total of five factors were extracted, with a cumulative variance contribution of 83.14%. The communalities of the items ranged from 0.620 to 0.890, indicating that the factor structure exhibited explanatory power.

To further validate the number of factors, a parallel analysis was conducted. The analysis generated 1,000 random data matrices matching the original sample size and number of variables, using the 95th percentile eigenvalues as the benchmark. As shown in Table 8, the actual eigenvalues of the first five factors exceeded the corresponding 95th percentile random eigenvalues, while the sixth factor did not. The parallel analysis results were fully consistent with the Kaiser criterion, supporting the five-factor structure.

**Table 8:** Comparison of eigenvalues between the actual sample and random data.

Factor	Actual eigenvalue	Mean random eigenvalue	95th percentile random eigenvalue
1	4.98	1.42	1.51
2	2.73	1.31	1.39
3	1.91	1.22	1.30
4	1.55	1.14	1.22
5	1.30	1.07	1.15
6	0.93	1.02	1.08

The rotated factor loading matrix was presented in Table 9. All items were clearly assigned to the five preset dimensions: Factor 1 corresponded to operational skills, factor 2 to higher-order thinking, factor 3 to metacognitive reflection, factor 4 to cognitive foundation, and factor 5 to ethical responsibility. In addition, the five factors were moderately positively correlated (*r* = 0.346 ~ 0.642), indicating that the dimensions were interrelated but not highly overlapping. These results indicate that the quality of the scale items is satisfactory.

**Table 9:** Results of exploratory factor analysis (rotated component matrix).

Dimension	Item	Factor 1	Factor 2	Factor 3	Factor 4	Factor 5
Operational skills	P4	**0.812**	0.245	0.132	0.108	0.089
	P5	**0.785**	0.198	0.156	0.124	0.076
	P6	**0.756**	0.223	0.145	0.098	0.092
Higher-order thinking	P7	0.234	**0.798**	0.187	0.112	0.098
	P8	0.187	**0.776**	0.156	0.134	0.087
	P9	0.212	**0.743**	0.198	0.098	0.105
Metacognitive reflection	P10	0.156	0.178	**0.805**	0.123	0.096
	P11	0.134	0.156	**0.778**	0.145	0.088
	P12	0.167	0.189	**0.752**	0.112	0.103
Cognitive foundation	P1	0.112	0.098	0.123	**0.821**	0.078
	P2	0.098	0.124	0.109	**0.793**	0.092
	P3	0.134	0.087	0.145	**0.764**	0.086
Ethical responsibility	P13	0.089	0.098	0.092	0.087	**0.838**
	P14	0.076	0.112	0.087	0.094	**0.816**
	P15	0.098	0.087	0.103	0.078	**0.789**

Bold values represent the highest factor loadings for each item, indicating the factor to which the item is assigned.

#### 5.2.3 Confirmatory factor analysis

Confirmatory factor analysis was conducted on the remaining 154 valid questionnaires (Dataset B) to examine the robustness of the five-factor structure identified in the exploratory factor analysis. The skewness values for all items ranged from 0.423 to 1.234, and the kurtosis values ranged from 0.281 to 1.863, indicating approximate univariate normality (Hoyle, 1995). Multivariate normality was assessed using Mardia’s coefficient. The critical ratio was less than 1.960 and did not reach statistical significance, which indicates that the data satisfy the multivariate normality assumption. Accordingly, maximum likelihood estimation was employed for parameter estimation.

##### 5.2.3.1 First-order confirmatory factor analysis

To test graduate students’ core generative AI literacy consists of five dimensions, one-factor, two-factor, three-factor, and four-factor models were constructed and compared. Model fit was evaluated using widely accepted criteria: *χ*^2^/df < 5, RMSEA < 0.080, SRMR < 0.050, CFI > 0.900, TLI > 0.900. Results are presented in Table 10. The five-factor model demonstrated significantly better fit than the competing models. CFI = 0.934, TLI = 0.919, and SRMR = 0.048 all met the recommended thresholds. RMSEA = 0.083 (90% CI [0.061,0.107]) slightly exceeded the 0.080 criterion. According to Maccallum et al. (1999), RMSEA values ranging from 0.080 to 0.100 signify mediocre model fit. Thus, the RMSEA value was within an acceptable range, though further refinement remained possible.

**Table 10:** Goodness-of-fit indices for competing confirmatory factor analysis models.

Dimension	*χ* ^2^	df	*χ*^2^/df	RMSEA	SRMR	CFI	TLI
One-factor model	682.45	90	7.58	0.207	0.168	0.568	0.512
Two-factor model	524.38	89	5.89	0.179	0.145	0.654	0.602
Three-factor model	398.76	87	4.58	0.153	0.126	0.735	0.698
Four-factor model	287.23	86	3.34	0.123	0.098	0.824	0.792
Five-factor model	170.48	83	2.05	0.083	0.048	0.934	0.919

One-factor model: all items loaded onto a single general factor. Two-factor model: cognitive foundation, operational skills, higher-order thinking, and metacognitive reflection were combined into one factor, with ethical responsibility as separate factor. Three-factor model: cognitive foundation, operational skills, and higher-order thinking were combined into one factor, with metacognitive reflection and ethical responsibility as separate factors. Four-factor model: cognitive foundation and operational skills were combined into one factor, with higher-order thinking, metacognitive reflection, and ethical responsibility as separate factors. Five-factor model: cognitive foundation, operational skills, higher-order thinking, metacognitive reflection, and ethical responsibility were treated as five distinct factors.

Further examination of the model’s internal fit quality indicated that the standardized factor loadings of all 15 items on their respective first-order factors ranged from 0.741 to 0.846, all exceeding the recommended threshold of 0.700 (Table 11). The inter-factor correlations ranged from 0.352 to 0.638, showing moderate positive correlations, suggesting that the five dimensions were interrelated yet discriminable. Standardized residual analysis showed that all absolute residual values were below the conventional threshold of 2.00, indicating that the model adequately reproduced the observed covariance structure without serious local misspecification. No modification indices were used for data-driven *post hoc* adjustments during model comparison and revision; all competing models were specified based on theoretical rationales. In summary, the five-factor model outperformed the other four competing models, supporting the reasonableness of conceptualizing graduate students’ generative AI literacy as a five-dimensional structure.

**Table 11:** Parameter estimates of the second-order confirmatory factor analysis model.

First-order factor	Standardized second-order path coefficient	Item	Standardized factor loading	Standard error	R^2^
Cognitive foundation	0.784	P1	0.812	0.035	0.659
		P2	0.787	0.036	0.619
		P3	0.765	0.038	0.585
Operational skills	0.762	P4	0.823	0.034	0.677
		P5	0.791	0.036	0.626
		P6	0.758	0.039	0.575
Higher-order Thinking	0.827	P7	0.803	0.035	0.645
		P8	0.776	0.037	0.602
		P9	0.741	0.040	0.549
Metacognitive reflection	0.749	P10	0.815	0.034	0.664
		P11	0.782	0.037	0.612
		P12	0.753	0.039	0.567
Ethical responsibility	0.713	P13	0.846	0.032	0.716
		P14	0.813	0.034	0.661
		P15	0.779	0.036	0.607

##### 5.2.3.2 Second-order confirmatory factor analysis

To examine whether the five first-order factors collectively reflect a higher-order construct of “graduate students’ generative AI literacy”, a second-order confirmatory factor model was constructed. The fit indices of the second-order model were: *χ*^2^/df = 2.10, RMSEA = 0.078 (90% CI [0.058, 0.098]), SRMR = 0.049, CFI = 0.935, TLI = 0.921, all meeting the recommended criteria, indicating good model fit. The fit of the second-order model was largely comparable to that of the five-factor correlated model in Table 10, supporting the existence of a higher-order “generative AI literacy” construct.

The convergent validity of the model was assessed by examining the standardized path coefficients between the second-order factor and each first-order factor. As shown in Table 11, the standardized path coefficients from the second-order factor to cognitive foundation, operational skills, higher-order thinking, metacognitive reflection, and ethical responsibility were 0.784, 0.762, 0.827, 0.749, and 0.713, respectively, all exceeding the recommended threshold of 0.700. The factor loadings of the items on their respective first-order factors ranged from 0.741 to 0.846, all statistically significant. These results indicate that the five dimensions effectively converge onto a single higher-order construct, demonstrating satisfactory convergent validity and further supporting the conceptualization of “generative AI literacy” as an overarching framework integrating the five dimensions.

#### 5.2.4 Reliability and validity testing

Reliability was first assessed. A reliability coefficient above 0.700 is generally considered to indicate good internal consistency (Zhu et al., 2025). As shown in Table 12, the Cronbach’s α coefficients for the five dimensions ranged from 0.796 to 0.842, and the McDonald’s ω coefficients ranged from 0.831 to 0.848. All exceeded the 0.700 threshold, indicating good internal consistency reliability across all dimensions of the scale.

**Table 12:** Internal consistency reliability coefficients.

Reliability coefficient	Cognitive foundation	Operational skills	Higher-order thinking	Metacognitive reflection	Ethical responsibility
Cronbach’s α	0.804	0.821	0.842	0.796	0.813
McDonald’s ω	0.831	0.841	0.848	0.831	0.846

Validity was then assessed, focusing on two aspects: construct validity and discriminant validity. Construct validity reflects the extent to which the trait structure measured by the scale aligns with the theoretical framework. Convergent validity was evaluated using the average variance extracted (AVE) and composite reliability (CR), following the criteria of Fornell and Larcker (1981): AVE > 0.500 and CR > 0.700. As shown in Table 13, the AVE values for all five dimensions exceeded 0.500, and the CR values all exceeded 0.700, indicating good internal consistency among the measurement indicators for each dimension and satisfactory convergent validity.

**Table 13:** Convergent validity of the graduate students’ generative AI literacy.

Convergent validity index	Cognitive foundation	Operational skills	Higher-order thinking	Metacognitive reflection	Ethical responsibility
AVE	0.517	0.538	0.551	0.501	0.524
CR	0.763	0.777	0.786	0.752	0.768

Discriminant validity aimed to examine the conceptual distinctiveness of the dimensions. Previous research had noted that relying solely on the Fornell-Larcker criterion might lack sufficient sensitivity in detecting discriminant validity issues (Henseler et al., 2015). Therefore, this study additionally employed the heterotrait-monotrait ratio (HTMT) as a Supplementary material to cross-validate the discriminant validity of the scale.

First, the Fornell-Larcker criterion was applied: discriminant validity was established if the square root of the AVE for each dimension was greater than its correlations with other dimensions (Fornell and Larcker, 1981). As shown in Table 14, the square root of the AVE for each dimension exceeded the corresponding inter-dimension correlations, and all correlations were below 0.850, indicating no serious multicollinearity issues. These results suggested that the scale exhibits strong discriminant validity. Further analysis revealed that the correlation coefficients between each pair of the five dimensions were significantly positive (*p* < 0.01), indicating that these five dimensions of literacy are not isolated from one another within the structure of graduate students’ generative AI literacy but rather exhibit interrelationships.

**Table 14:** Discriminant validity of the graduate students’ generative AI literacy.

Dimension	Cognitive foundation	Operational skills	Higher-order thinking	Metacognitive reflection	Ethical responsibility
Cognitive foundation	(0.719)				
Operational skills	0.527***	(0.733)			
Higher-order thinking	0.486***	0.642***	(0.742)		
Metacognitive reflection	0.412***	0.468***	0.537***	(0.708)	
Ethical responsibility	0.346***	0.384***	0.423***	0.459***	(0.724)

*** *p* < 0.001, ** *p* < 0.01, * *p* < 0.05; values in parentheses represent the square roots of the AVE for each factor.

Second, the HTMT ratio was used to further assess discriminant validity. HTMT values below 0.850 indicate adequate discriminant validity between two constructs (Voorhees et al., 2016). As shown in Table 15, all HTMT values for each pair of dimensions were below the threshold of 0.850, providing evidence that the five dimensions demonstrated adequate discriminant validity.

**Table 15:** HTMT ratio matrix for the five dimensions.

Dimension	Cognitive foundation	Operational skills	Higher-order thinking	Metacognitive reflection	Ethical responsibility
Cognitive foundation					
Operational skills	0.614				
Higher-order thinking	0.563	0.726			
Metacognitive reflection	0.481	0.542	0.627		
Ethical responsibility	0.402	0.447	0.495	0.531	

## 6 Conclusions and implications

### 6.1 Research conclusions

This study constructed and validated a structural model of graduate students’ generative AI literacy. The main conclusions were as follows.

Graduate students’ generative AI literacy comprised five dimensions: cognitive foundation, operational skills, higher-order thinking, metacognitive reflection, and ethical responsibility. This structure was validated through both grounded theory analysis and questionnaire testing. It moved beyond a narrow focus on basic AI operations by integrating technical execution, cognitive processing, self-monitoring, and ethical judgment into a coherent framework, reflecting a theoretical progression from tool use to literacy formation. Each dimension included three second-level indicators, totaling 15 indicators. The scale developed on this basis measured graduate students perceived literacy rather than their objective performance in authentic academic tasks. Results from confirmatory factor analysis, reliability testing, and validity testing indicated that the scale demonstrated acceptable structural validity and internal consistency, supporting its use as a measure of perceived generative AI literacy among graduate students.

Based on the grounded theory analysis, cognitive foundation provided the underlying knowledge base for graduate students’ use of AI tools, while higher-order thinking was responsible for critically evaluating, synthesizing, and deeply processing AI-generated content. Operational skills served as the practical tool that translated cognitive and critical thinking capacities into academic outputs. Metacognitive reflection continuously performed monitoring, evaluation, and regulatory functions, ensuring that graduate students maintained academic autonomy throughout their use of AI. Ethical responsibility provided value guidance and normative direction for the functioning of all dimensions. The five dimensions worked in synergy to jointly support graduate students in achieving academic breakthroughs in human-AI collaboration and in completing the transition from AI users to academic leaders.

### 6.2 Research limitations

This study has several limitations.

First, the sample sources were limited. All interviewees were professors whose systematic professional judgments regarding graduate education objectives and competency requirements were inevitably shaped by normative expectations. Therefore, their perspectives might not fully reflect graduate students’ authentic experiences in academic practice. The samples were geographically concentrated and predominantly male, with a relatively small sample size, which further limited the generalizability of the findings to other regions or cultural contexts. Future research should expand geographic and gender coverage and incorporate interview data from graduate students’ perspectives to enrich the model.

Second, the measurement scale was in a preliminary stage of development. The 15 s-level indicators were each measured by a single item, which precluded the estimation of internal consistency and failed to capture the multidimensionality of the constructs. This scale should therefore be considered a proof-of-concept instrument rather than a fully validated measure. Future research should develop multiple items per dimension, incorporate performance-based assessments, and conduct test–retest reliability checks so as to establish stability and criterion-related validity. Measurement invariance should also be tested across gender, educational level, discipline, and institution type, along with cross-cultural applicability.

Third, this study relied on cross-sectional, single-source self-report data, which may introduce common method bias. Such data could neither capture the transition from AI users to autonomous researchers nor reflect actual AI literacy in authentic tasks due to potential social desirability bias. Furthermore, the examination of inter-dimensional relationships was based primarily on correlation analyses and a second-order factor model, which were insufficient to establish dynamic synergies or causal ordering among the five dimensions. Future research should employ longitudinal designs, configurational analysis, or multi-group comparisons to test the synergistic mechanisms and discriminant validity among the dimensions.

### 6.3 Research implications

The above findings reveal the multidimensional structure of graduate students’ generative AI literacy. To translate this theoretical framework into actionable cultivation strategies, the following aspects can be pursued.

Enhance cognitive foundation and operational skills by establishing a dual-track cultivation system integrating curriculum and project-based learning. Develop a tiered curriculum: junior graduate students should focus on the cognitive foundation of generative AI, encompassing understanding of technical principles, recognition of tool capabilities, and identification of application scenarios; senior graduate students should emphasize operational skills of generative AI, including prompt design, multi-turn dialogue, and output processing. In terms of project-based practice, develop interdisciplinary project courses that integrate the cognitive foundation and operational skills of generative AI into authentic research tasks, enabling graduate students to transform knowledge reserves into practical competencies.

Higher-order thinking and metacognitive reflection should be cultivated to facilitate the transition from tool use to academic leadership. Universities should adopt case-based discussions and academic writing workshops to guide graduate students in critically evaluating AI-generated content for logical and factual validity, leveraging AI to expand research perspectives and generate independent hypotheses, and clarifying the boundaries between human and AI task allocation. Concurrently, graduate students should be guided to continuously monitor the appropriateness of their operations during AI use, evaluate the effectiveness of AI assistance, and develop a metacognitive cycle of “thinking–action–reflection–optimization,” thereby maintaining cognitive autonomy while benefiting from AI-enhanced efficiency.

Strengthen the bottom line of ethical responsibility by integrating AI ethics education throughout graduate training. Ethical responsibility serves as the value baseline for graduate students to conduct responsible research in human-AI collaboration. Universities should offer specialized courses on AI ethics organized around three dimensions: awareness of academic integrity, awareness of academic social responsibility, and awareness of knowledge contribution attribution. Through training in academic norms, graduate students should be guided to clarify the boundaries and attribution requirements of AI assistance, enhance their awareness of intellectual property and citation standards, and cultivate their self-awareness and judgment in upholding academic integrity in the AI era.

Provide training for supervisors to help them model appropriate AI use and guide students in developing critical and ethical AI literacy. Supervisors play a pivotal role in shaping students’ attitudes toward AI, as graduate students often emulate their mentors’ practices and internalize their standards. Therefore, supervisor training programs should cover not only the technical operation of generative AI tools but also strategies for integrating AI literacy cultivation into daily research supervision, such as discussing ethical dilemmas, reviewing AI-assisted writing together, and encouraging metacognitive reflection on AI use. This creates a supportive environment for the social transmission of literacy skills, where norms and competencies are co-constructed through ongoing supervisor-student interaction.

## Data Availability

The raw data supporting the conclusions of this article will be made available by the authors, without undue reservation.
