# Supplementary material for: Modeling and measuring graduate students’ generative AI literacy: a study based on Marzano’s taxonomy
Source: Front Psychol. 2026 Sep 17;17:1883978. doi: 10.3389/fpsyg.2026.1883978 (PMC13627062; doi:10.3389/fpsyg.2026.1883978)
Supplement: Supplementary file 1 [file Supplementary_file_1.docx]

**Dear Student,**

Thank you for participating in this survey. This questionnaire is designed to assess graduate students' generative AI literacy. Your responses will be used solely for academic research purposes and will remain strictly anonymous. There are no right or wrong answers. Please answer each item based on your actual situation and true feelings.

**I. Basic Information**

(Please mark “√” for your choice)

Gender: □ Male □ Female

Disciplinary Background: □ Science and Engineering □ Humanities and Social Sciences □ Agriculture □ Medicine □ Interdisciplinarity

Degree Level: □ Master’s Degree Students □ Doctoral Degree Students

University Type: □ “Double First-Class” Universities □ Other Universities

**II. Formal Scale**

Please rate each statement according to your actual situation and true feelings using a 5‑point Likert scale:1 = Strongly Disagree2 = Disagree3 = Neutral4 = Agree5 = Strongly Agree

|  | Item | Strongly Disagree | Disagree | Neutral | Agree | Strongly Agree |
| --- | --- | --- | --- | --- | --- | --- |
| 1 | I understand the basic working principles of large language models, as well as their inherent limitations in factual generation, logical reasoning, and knowledge representation. |  |  |  |  |  |
| 2 | I am able to compare the functional features, strengths, and weaknesses of different generative AI tools, and make reasonable choices based on my academic task requirements. |  |  |  |  |  |
| 3 | I can accurately determine the scope of generative AI use in academic work, and recognize the differences in the degree of AI reliance across various tasks. |  |  |  |  |  |
| 4 | I can design effective prompts, such as setting AI's academic role, clearly describing research tasks, specifying output format, and guiding AI to reason step by step. |  |  |  |  |  |
| 5 | I can engage in in-depth interaction with generative AI through multi-turn questioning, rephrasing inquiries, and correcting AI misunderstandings. |  |  |  |  |  |
| 6 | I am able to filter, organize, restructure, and optimize AI-generated content, and integrate it organically with my own ideas. |  |  |  |  |  |
| 7 | I can comprehensively examine and verify the content generated by generative AI, including its logical soundness, argumentative rigor, potential biases, as well as possible issues such as fabricated literature, incorrect data, temporal inconsistencies, or false citations. |  |  |  |  |  |
| 8 | I can use generative AI to broaden my research ideas, discover interdisciplinary connections, and generate innovative research hypotheses. |  |  |  |  |  |
| 9 | I can reasonably design the division of labor between human and AI, clarifying which tasks should be assisted by AI and which should be led by myself. |  |  |  |  |  |
| 10 | When using generative AI, I am able to monitor whether my operations are appropriate, and guard against over-reliance or cognitive inertia. |  |  |  |  |  |
| 11 | I can objectively evaluate the actual effectiveness of generative AI-assisted academic work from multiple dimensions, including time investment, output quality, and cognitive load. |  |  |  |  |  |
| 12 | I am able to transfer AI use strategies developed in one academic task to other task contexts, and adjust them flexibly according to the actual situation. |  |  |  |  |  |
| 13 | When using generative AI, I comply with academic norms, clearly indicate the scope of AI use, and avoid academic misconduct. |  |  |  |  |  |
| 14 | I pay attention to the impact of generative AI on the academic ecosystem and social equity, and am willing to assume corresponding academic social responsibilities. |  |  |  |  |  |
| 15 | I can clearly distinguish between human and AI contributions in knowledge production, properly acknowledge sources, and respect intellectual property rights. |  |  |  |  |  |
